# A cross-sectional online survey on oncologists’ attitudes toward and experiences with providing patients with audio recordings of their medical encounters

**DOI:** 10.1038/s41598-025-01962-8

**Published:** 2025-05-16

**Authors:** Cheyenne Topf, Pola Hahlweg, Isabelle Scholl

**Affiliations:** Department of Medical Psychology, University Medical Center Hamburg-Eppendorf, Hamburg, Germany

**Keywords:** Oncology, Consultation recordings, Cross, Cross-sectional online study, Patient-centered care, Patient information, Patient–physician-relationship, Patient education, Health care, Oncology, Cancer

## Abstract

Providing patients with audio recordings of their medical encounters, termed consultation recordings, has shown promising benefits, especially for patients with cancer. While patients favor these recordings, international research indicated that physicians have mixed feelings. To date, research specific to Germany remains limited. This study investigated German oncologists’ attitudes and experiences through a nationwide cross-sectional quantitative online survey, informed by semi-structured interviews. Attitudes, prior experiences and desire for consultation recordings were assessed. Data was analyzed using descriptive statistics and subgroup analyses. Ninety-four physicians participated, with about half expressing a generally positive attitude, though overall attitudes were mixed. Expected benefits included improved patient recall and communication, while concerns centered around potential misuse of recordings, data confidentiality, and increased legal risks. Experiences were limited, with 12% reporting previous use. Fourteen percent expressed a willingness to offer recordings in future and 31% were undecided. This study highlights cautious openness among German oncologists, tempered by concerns over data security and legal implications, which may hinder adoption. This could be addressed by providing sound evidence regarding benefits and concerns, and enabling positive experiences. Further research should include feasibility testing in routine cancer care and re-evaluation of these results in representative samples and other specialties.

**Supplementary Information:**

The online version contains supplementary material available at 10.1038/s41598-025-01962-8.

## Introduction

Patients with cancer are often confronted with complex and extensive information^1^. More often than not, their emotional reactions interfere with the cognitive processing of those information^2,3^. Research indicates that up to 80% of information provided during medical encounters cannot be accurately recalled by patients^3–5^. One internationally well researched intervention to address this issue is the provision of audio recordings of their medical encounters to patients, commonly referred to as consultation recordings^2,4,6,7^. There is evidence that these recordings offer a range of benefits: they help patients recall information^2,4,6–11^, enhance understanding^6,8,10,12^, and increase the feeling of being informed^9^. Furthermore, they can empower patients^10,11,13,14^, facilitate discussions with family members^9–11,14^, improve treatment decision-making^9,10,15^, reduce anxiety and depression^2,7,15^, and improve overall satisfaction with care^9^.

While the benefits for patients are well-documented, the implications for physicians are more ambiguous. International research suggests that physicians’ views on consultation recordings are shaped by their professional experience and past positive encounters with such recordings^12,16,17^. Additionally, results from a previous study indicate a relation between greater clinical experience and higher physician age with more positive attitudes toward consultation recordings^16^. Physicians acknowledge the above-mentioned benefits of consultation recordings^6,12,16–19^, but they express unease about being recorded. They worry about confidentiality and data protection^17,20^, legal implications^6,12,16,17,19,20^, and recordings being misused or taken out of context^12,16,17,20^. There is also apprehension about a negative impact on the patient-physician relationship^6,12,16,17,19,20^. Furthermore, some physicians worry that consultation recordings might escalate patient anxiety^19^. Practical concerns include that consultation recordings could prolong consultations^2,19,20^. Prior studies have shown mixed results regarding consultation duration^2,11,21,22^ and patients provided with recordings may require fewer follow-up consultations^2,11,21^.

There are different ways of implementing consultation recording, including patient-led (i.e., patients ask clinicians for permission to record with their own devices), covert, and service- or hospital-led (i.e., the hospital or physician provides the recording, possibly through smartphone apps or patient portals) recordings^23^. Despite these options, the prevalence of consultation recordings remains low. In a previous study in Germany only 5% of participating patients with cancer indicated prior recording experience^24^. Studies from the United Kingdom and the United States indicated that 15^13^ to 18%^6^ of patients had recorded at least one medical encounter. In contrast, up to 93% of physicians reported having experienced consultation recordings^6,12,16^.

While the majority of patients has been found to express positive attitudes toward consultation recordings^6,9,11,15,20,24,25^, physicians have reported mixed feelings^2,6,7,12,16–20^ and research about physicians in the German healthcare context is lacking. However, scientific evidence is essential to address physicians’ concerns and facilitate implementation^12,26^. Given that the effective implementation of interventions heavily depends on contextual factors such as culture of healthcare delivery^27^, and considering the limited research on consultation recordings in Germany, we aimed to assess the attitudes and experiences of oncologists in Germany toward the provision of consultation recordings. Our goal was to evaluate the feasibility of such recordings in the German healthcare context.

## Methods

We report on a cross-sectional nationwide quantitative online survey exploring attitudes and experiences regarding consultation recordings of physicians working with patients with cancer in Germany. Reporting follows the Checklist for Reporting Results of Internet E-Surveys (CHERRIES)^28^ (cp. Supplementary file 1). The methodology described below parallels that of a previous study we conducted with patients with cancer^24^.

### Participants

Physicians working in oncology were eligible to participate. For pragmatic reasons, we planned to include 100 participants in the quantitative survey. We did not perform an a priori power analysis since predominantly descriptive data analyses were planned.

### Materials and questionnaires

Semi-structured qualitative interviews with ten oncologists were conducted to inform the development of the quantitative questionnaire. The interviews followed a guideline with questions about participants’ experiences with consultation recordings and their attitudes toward them. Participants were recruited using convenience sampling. The interviews were conducted between February 2022 and March 2022, and were subsequently audio-recorded, transcribed, and analyzed using qualitative content analysis^29^. Additional information on the qualitative methods and results can be found in Supplementary File 2. Furthermore, the development of the quantitative questionnaire was based on qualitative interviews with people from the general public in an unpublished preliminary study by the authors, on the integrative model of patient-centeredness^30^, and additional literature^8,13,18,19,31,32^. The integrative model of patient-centeredness was specifically used to incorporate aspects related to patient-centeredness^30^. The analysis of qualitative interviews from our previous unpublished study and from this study informed the survey regarding aspects that influence participants’ attitudes toward consultation recordings. The survey assessed participants’ experience with, attitudes toward, and desire for consultation recordings. It was pretested with physicians from the study’s advisory board, colleagues from our department, and people from the general population (n = 10). The final survey can be found in Supplementary File 3.

The first pages of the survey included the informed consent and described what consultation recordings are (i.e. the provision of audio recordings of medical encounters). In the subsequent first part, participants were asked about their experiences with consultation recordings. If they indicated prior experiences with such recordings, we asked them further questions about it (e.g., “Who suggested making the audio recording?”, “How was the audio recording made?”). In the second part, we assessed their general attitude toward consultation recordings with three items (see Supplementary File 3 for items). Each item was scored on a 6-point Likert scale, ranging from *does not apply at all (*= *1)* to *fully applies (*= *6)* or from *very negative (*= *1)* to *very positive (*= *6).* In addition, participants assessed 50 statements about possible benefits and concerns regarding consultation recordings on a 6-point Likert scale ranging from *completely disagree (*= *1)* to *completely agree (*= *6)*. The third part included questions about their desire for future consultation recordings. If participants indicated that they were open to record medical encounters in the future, they were asked further questions (e.g., “Would you be open to patients recording on their cell phone?”). For five items, participants were able to further specify their answer in an open format (mostly, if their answer was not yet included).

In addition, participants’ preferred role in treatment decisions was assessed with an adapted version of the Control Preferences Scale (CPS)^33^. The one-item instrument measures whether participants prefer the decision-making to be led by the physician, the patient, or shared. Participants’ proclivity for actively engaging with technical systems was assessed by the German version of the Affinity for Technology Interaction Short Scale (ATI-S)^34^. ATI-S consists of four items using a 6-point Likert scale, ranging from *completely disagree (*= *1) to completely agree (*= *6)*. ATI-S showed high McDonald’s omega, factor loadings, item difficulty and discrimination, and construct validity^34^. Furthermore, we collected demographic data (e.g. age, gender), work experience (e.g. residency training, experience in oncology), and knowledge about laws regulating audio recordings in Germany.

### Data collection

We followed a convenience sampling method, using several recruitment strategies. We (a) invited participants via email by contacting more than five-hundred oncological in- and outpatient clinics (including all Comprehensive Cancer Centers nationwide) and working groups of a professional oncological society; (b) distributed study invitations via social media; and (c) handed out leaflets at various conferences. Further details on the recruitment process can be found in Supplementary File 4. Participants were required to provide informed consent electronically, before participating in the online survey. They confirmed through self-report that they are treating patients with cancer. The survey was initially conducted between June 2022 and April 2023. As we were unable to reach our target sample size of 100 participants, we added a second survey period from October 2023 to April 2024. We utilized the LimeSurvey platform. Participants had the opportunity to receive a 10 Euro incentive.

### Data analysis

We only included participants in the dataset for analysis who met the inclusion criteria and completed the survey. For quality assurance, we assessed survey completion times and inconsistent responses^35^. These measures were chosen after having identified a delayed influx of a conspicuously high number of consecutive participations within a short period of time during data preparation. We set the minimal reasonable response time to 2 s per item, leading to a minimal survey completion time of 2.67 min (80 items × 2 s). Inconsistent responses were assumed when 5 or more consecutive participants from different geographical locations (i.e. not from the same institution) indicated an unlikely extent of experience with consultation recordings. They led to the exclusion of 8 cases (n = 0 cases due to short completion times, n = 8 cases due to logically inconsistent responses). Missing data was not imputed. The ATI-S was analyzed according to its manual. Open-format specifications to items were categorized and subsequently included in the analyses.

We analyzed the data using SPSS 27. For all items, we calculated descriptive statistics. In addition, we formulated two hypotheses for subgroup testing, based on results from a study by Jimenez and colleagues^16^: (1) The attitude toward consultation recordings is more positive with more work experience; (2) The attitude toward consultation recordings is more positive with older physician age. Regarding hypothesis 1, we compared four groups with different years of work experience: “less than 5 years”, “5 to 10 years”, “11 to 20 years”, and “more than 20 years”. Regarding hypothesis 2, we compared three age groups: “18 to 39 years”, “40 to 59 years”, and “60 years and older”. The Jonckheere Terpstra test, a rank-based nonparametric test, was employed to determine whether there is a statistically significant trend between the ordinal independent variables (years of work experience, age groups) and the ordinal dependent variable (general attitude)^36^. Given the presence of two co-primary hypotheses, significant level was set to *p* < 0.025.

## Results

### Qualitative results

Ten oncologists participated in the qualitative study to develop the quantitative questionnaire. Of those, 8 were male (80%) and 2 were female (20%). Seven had completed their residency (70%) and 3 were in residency training (30%). Most were working at a hospital (n = 6, 60%) and 4 (40%) had less than 5 years, 1 (10%) 5 to 10 years, 2 (20%) 11 to 20 years, and 3 (30%) more than 20 years of experience in oncology. Participants were found to have prior experiences with consultation recordings in some cases and reported a wide range of aspects regarding their attitudes toward consultation recordings. Additional results are presented in Supplementary File 2.

### Quantitative results

#### Participant characteristics

**Fig. 1:**
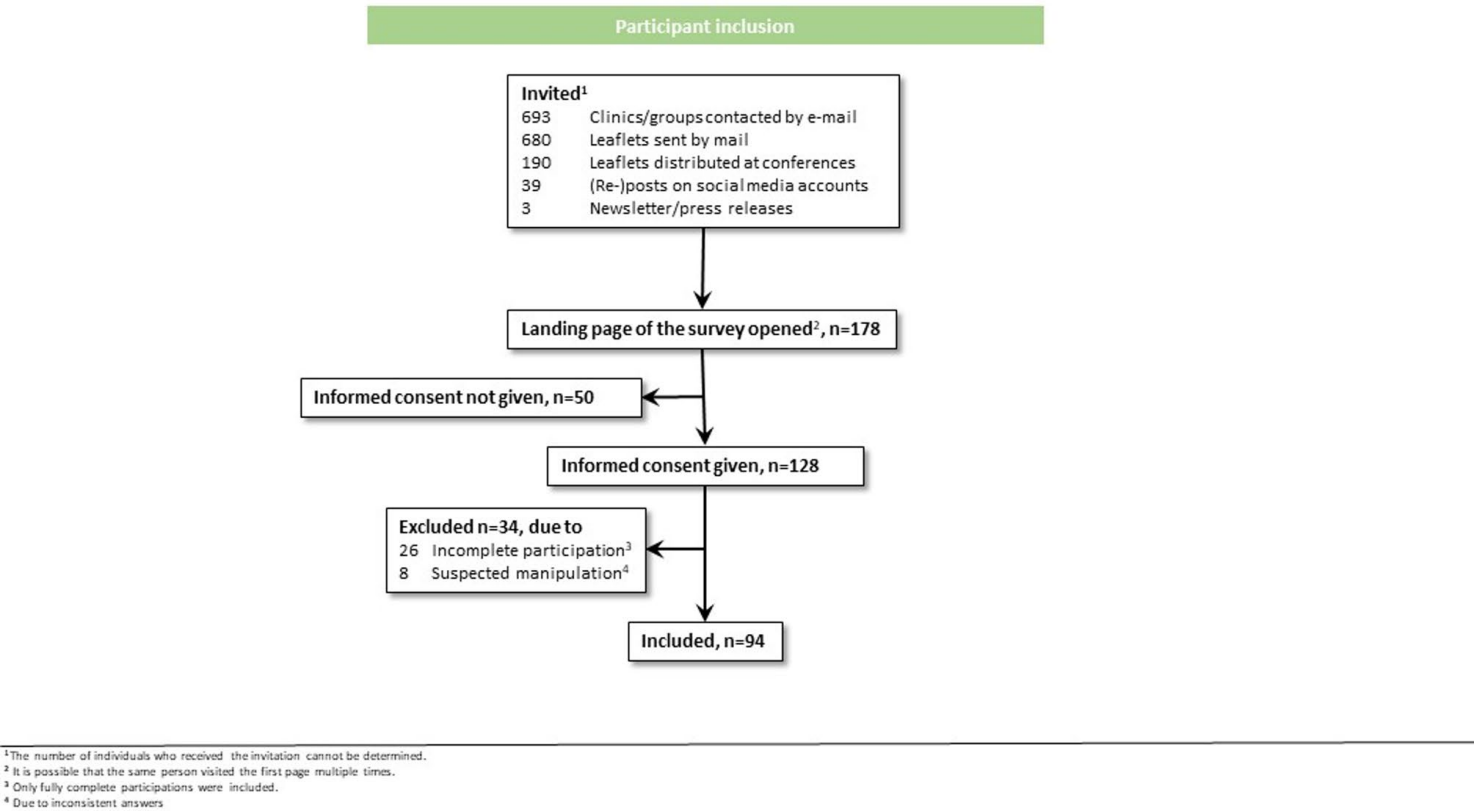
Participant inclusion.

One-hundred seventy-eight individuals accessed the survey’s initial page, and out of these, 128 consented to participate. We removed 26 participants for incomplete submissions and an additional eight due to suspected manipulation. Ninety-four participants met the inclusion criteria and completed the survey (see Fig. 1). About half of the participants were male (n = 50, 53.2%). Most participants were between 30 to 39 years (n = 32, 34%) and 40 to 49 years (n = 29, 30.9%) old. Twenty-seven (28.7%) had less than 5 years, 19 (20.2%) 5 to 10 years, 21 (22.3%) 11 to 20 years, and 27 (28.7%) more than 20 years of work experience in oncology. Thirty participants (31.9%) were in residency training and 64 (68.1%) had completed their residency. Most of the participants worked at a hospital, either as junior physicians (n = 35, 37.2%) or as senior or head physicians (n = 32, 34%). The sample showed a moderate level of technological affinity (X̅ = 3.78; SD = 1.21). For reference, the average technological affinity in a German quota sample is 3.61 (SD = 1.08)^37^. Most participants did not know the laws regulating audio recordings in Germany (n = 80, 85.1%) and the vast majority of participants preferred a patient-led (n = 51, 54.3%) or shared (n = 39, 41.5%) decision-making style. Additional participant characteristics can be found in Table 1.

**Table 1:** Participant characteristics.

Participant characteristics (N = 94)	N (%)
Sex	
Female	44 (46.8)
Male	50 (53.2)
Age group	
18–29 years	9 (9.6)
30–39 years	32 (34.0)
40–49 years	29 (30.9)
50–59 years	13 (13.8)
60–69 years	10 (10.6)
70 years and older	1 (1.1)
Work experience in oncology	
Less than 5 years	27 (28.7)
5–10 years	19 (20.2)
11–20 years	21 (22.3)
More than 20 years	27 (28.7)
Affinity for Technology Interaction (range 1 to 6, higher more)^1^	
Mean (SD)	3.78 (1.21)
Range	1.25–6
Education level	
In residency training^1^	30 (31.9)
Completed residency	64 (68.1)
Perceived knowledge of laws regulating audio recordings in Germany	
Yes	10 (10.6)
No	80 (85.1)
Current position / work setting	
Junior physician at a hospital	35 (37.2)
Senior or head physician at a hospital	32 (34.0)
Working in own outpatient practice	11 (11.7)
Employed in an outpatient practice	7 (7.4)
Other	9 (9.6)
Preferred level of involvement in treatment decisions	
Patients should make the decision what medical treatment they receive	12 (12.8)
Patients should ultimately make the decision about their medical treatment, after having seriously considered my medical opinion	39 (41.5)
I prefer my patient and I to share the responsibility for making the decision which medical treatment is best for them	39 (41.5)
I prefer to make the final decision about the patient’s medical treatment, considering their opinion	4 (4.3)
As a doctor, I prefer to make all decisions concerning the patient’s medical treatment	0 (0.0)
Work location (German states)^2^	
Hamburg	37 (39.4)
North Rhine-Westphalia	16 (17.0)
Baden-Württemberg	10 (10.6)
Bavaria	8 (8.5)
Lower Saxony	6 (6.4)
Schleswig–Holstein	5 (5.3)
Rhineland-Palatinate	4 (4.3)
Saxony-Anhalt	4 (4.3)
Saxony	2 (2.1)
Berlin	1 (1.1)
Brandenburg	1 (1.1)

Frequencies and percentages not adding up to the total number of participants indicate missing data; ^1^In Germany, physicians undergoing residency training in oncology and hematology regularly treat cancer patients; ^2^Items are ordered from highest to lowest frequency.

#### Attitudes toward the provision of consultation recordings

**Fig. 2:**
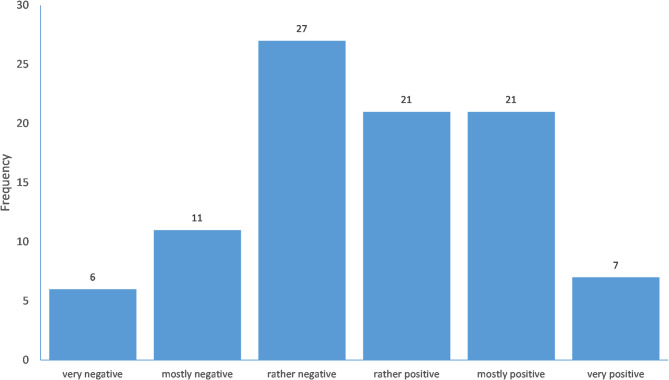
Attitude toward the provision of audio recordings of medical encounters for patients.

Participants’ general attitude toward consultation recordings (on a scale from very negative (= 1) to very positive (= 6)) had a mean value of 3.66 (SD = 1.33, Median = 4.00). Approximately half of the participants (n = 49, 52.7%) reported a rather positive, mostly positive, or very positive attitude toward consultation recordings (see Fig. 2). The item ”I would allow patients to make a consultation recording, but I would not proactively offer it” had a mean value of 3.88 (SD = 1.45, Median = 4.00) on a scale from does not apply (= 1) to fully applies (= 6). The item ”In principle, it would be okay for me to offer a consultation recording to patients” had a mean value of 3.94 (SD = 1.55, Median = 4.00) on the same scale as the previous item.

Table 2 presents various statements about potential benefits of consultation recordings and participants’ level of agreement on a scale from completely disagree (= 1) to completely agree (= 6), ranked from highest to lowest mean. Overall, participants showed moderate to high agreement with the proposed benefits of consultation recordings (means from 2.68 to 4.89, medians from 3.00 to 5.00). The following statements received highest agreement: Consultation recordings could improve recall of the information discussed (X̅ = 4.89; SD = 1.13; Median = 5.00), enhance preparation for follow-up appointments (X̅ = 4.41; SD = 1.35; Median = 5.00), and allow patients to share information with their relatives (X̅ = 4.31; SD = 1.20; Median = 4.00). Furthermore, consultation recordings could provide evidence of what was said and done (X̅ = 4.24; SD = 1.33; Median = 4.00) and improve understanding of information (X̅ = 4.23; SD = 1.39; Median = 4.00).

**Table 2:** Levels of agreement toward different statements about benefits of consultation recordings, thematically clustered^1,2^

	N	Mean (SD)	Median
Information Recall, Understanding and Communication			
A consultation recording…			
…allows patients to have a better recall of the information discussed	94	4.89 (1.13)	5.00
…enhances the understanding of information	94	4.23 (1.39)	4.00
…allows patients to retrospectively verify correct understanding of the information	94	4.21 (1.29)	4.00
…allows patients to ensure that the physician has understood them correctly	94	3.69 (1.28)	4.00
…improves the quality of communication	93	3.08 (1.27)	3.00
Patient Engagement and Empowerment			
A consultation recording…			
…allows patients to prepare for follow-up appointments (e.g. note down questions)	94	4.41 (1.35)	5.00
…allows patients to compare their treatment options and make the best decision	93	3.61 (1.23)	4.00
…encourages patients to engage with their diagnosis	93	3.56 (1.35)	4.00
…facilitates patients’ active and self-responsible managing of their disease	94	3.54 (1.78)	4.00
Engagement and Support of Relatives			
A consultation recording…			
…allows patients to share information with their relatives	94	4.31 (1.20)	4.00
…allows relatives to provide better support to the patient	92	3.91 (1.28)	4.00
(Patient) Safety and Protection			
A consultation recording…			
…provides evidence of what was said and done	93	4.24 (1.33)	4.00
…allows a better adherence to medical instructions	94	3.70 (1.14)	4.00
…provides evidence in case of malpractice	92	3.55 (1.47)	4.00
…provides protection for patients and physicians	92	3.01 (1.33)	3.00
Different Consultation Occasions			
A consultation recording…			
…is especially helpful in consultations in which treatment decisions are made	93	4.10 (1.25)	4.00
…is especially helpful when starting or changing a treatment	93	4.01 (1.33)	4.00
…is especially helpful in complex and lengthy treatments	92	3.88 (1.36)	4.00
…should also be conducted when the diagnosis is communicated during the consultation	92	3.71 (1.45)	4.00
…is helpful for treatment planning	93	3.17 (1.46)	3.00
…should be made even in brief consultations with little amount of new information	91	2.69 (1.10)	3.00
Different Groups of People			
A consultation recording…			
…is especially helpful for people with language barriers	92	3.74 (1.24)	4.00
…is especially helpful for people with cognitive deficits	94	3.21 (1.17)	3.00
…is especially helpful for older people	93	3.19 (1.29)	3.00
Patient-Physician Relationship			
A consultation recording…			
…facilitates an equal collaboration between patient and physician	93	3.28 (1.30)	3.00
…allows patients to share information with other healthcare professionals	94	3.10 (1.50)	3.00
…improves the trust between patients and physicians	90	3.09 (1.35)	3.00
…allows physicians to be more responsive of concerns and needs of patients	92	2.93 (1.32)	3.00
Organizational Benefits			
A consultation recording…			
… reduces consultation length	93	2.68 (0.97)	3.00

^1^Answers were assessed on a 6-point Likert scale, ranging from *completely disagree (*= *1)* to *completely agree (*= *6);* ^2^Items are ordered from highest to lowest mean.

Regarding concerns about consultation recordings, participants showed moderate to very high agreement with the proposed statements (means from 2.53 to 5.20, medians from 3.00 to 6.00; see Table 3). Primary concerns of the participants centered around the misuse of consultation recordings, for example that the recordings could be shared without permission (X̅ = 5.20; SD = 1.08; Median = 6.00), about confidentiality and data protection (X̅ = 4.99; SD = 1.27; Median = 5.00), and the handling of the recording (X̅ = 4.96; SD = 1.28; Median = 6.00). In addition, they were concerned that consultation recordings could be used as evidence against physicians (X̅ = 4.82; SD = 1.20; Median = 6.00) and that it would put pressure on physicians (X̅ = 4.76; SD = 1.24; Median = 5.00).

**Table 3:** Levels of agreement toward different statements about concerns regarding consultation recordings, thematically clustered^1,2^

	N	Mean (SD)	Median
Confidentiality and Data Protection			
I am concerned…			
… that consultation recordings could be passed on undesirably	92	5.20 (1.08)	6.00
…about confidentiality and data protection if consultations were recorded	93	4.99 (1.27)	5.00
…about what happens with the consultation recording	93	4.96 (1.28)	6.00
Evidence and Accountability			
I am concerned…			
…that a consultation recording would be used as evidence against physicians	92	4.82 (1.20)	5.00
…that a consultation recording would put pressure on physicians	94	4.76 (1.24)	5.00
…that the recording of the consultation could be distorted	93	4.45 (1.47)	5.00
Physician–Patient Relationship			
I am concerned…			
…that physicians would be reserved and less open if consultations were recorded	94	4.48 (1.27)	5.00
…that the physician–patient-relationship would be more formal if consultations were recorded	92	4.33 (1.29)	4.00
…that the trust between patients and physicians would decrease if consultations were recorded	88	3.58 (1.40)	3.00
…that patients would be reserved and less open if consultations were recorded	94	3.43 (1.27)	3.00
Impact on Patient Well-being			
I am concerned…			
… that patients overinterpret statements made by the physician on the recording	94	4.61 (1.12)	5.00
…that relatives could pressure patients into allowing them to listen to their consultation recording	94	4.36 (1.19)	4.00
…about patients perceiving a recording device as stressful during consultations	94	3.44 (1.40)	3.00
…that listening to the consultation recording would be a psychological burden for patients	92	3.15 (1.27)	3.00
…that a consultation recording puts too much responsibility on patients	94	2.53 (0.90)	3.00
Consultation Dynamics and Routines			
I am concerned…			
…that consultation recordings prolong consultations	92	3.63 (1.30)	3.00
…that the quality of the communication decreases through a consultation recording	90	3.53 (1.28)	3.00
…that a consultation recording disrupts clinical routines	93	3.28 (1.25)	3.00
(Technical) Feasibility			
I am concerned…			
…that recording consultations is too complicated for physicians	93	2.97 (1.44)	3.00
…that the technical requirements for making consultation recordings do not exist	92	2.97 (1.42)	3.00
…that recording consultations is too complicated for patients	93	2.86 (1.28)	3.00

^1^answers were assessed on a 6-point Likert scale, ranging from *completely disagree (*= *1)* to *completely agree (*= *6)*; ^2^Items are ordered from highest to lowest agreement.

#### Association between work experience and age with attitudes toward consultation recordings

Descriptively, participants with less than 5 years of work experience in oncology showed a Median of 4.00 regarding their general attitude toward consultation recordings, participants with 5 to 10 years of work experience of 4.00, participants with 11 to 20 years of work experience of 4.00, and participants with more than 20 years of work experience of 3.00. In the Jonckheere-Terpstra test, we found no statistically significant trend in the general attitudes with longer work experience in this sample (T_JT_ = 1611.000, z = 0.032, *p* < 0.975).

Regarding the age groups, the medians for the general attitude item in the three groups were as follows: Median = 4.00 for 18 to 39 years, Median = 4.00 for 40 to 59 years, and Median = 3.00 for older than 60 years. The Jonckheere-Terpstra test did not show a statistically significant trend in the general attitudes with rising age in this sample (T_JT_ = 1305.500, z = 0.109, *p* < 0.913).

#### Experience with consultation recordings

Eleven participants (11.7%) reported experiences with consultation recordings. Most of them (n = 9, 81.8%) had recorded on multiple occasions. They predominantly did not have access to the recording themselves (n = 7, 63.6%), but if so (n = 4, 36.4%), listened to the recording in half of the cases (n = 2, 50%). For the following items, multiple answers could apply. The recordings were either initiated by the patients (n = 6, 54.5%), their accompanying persons (n = 4, 36.4%), or by the physicians themselves (n = 5, 45.4%). One participant (9.1%) answered that it was standard practice at their workplace and another one (9.1%) that they did the consultation recording as part of a research project (i.e., subsequent study phase of this study). Recordings were done either with a cell phone (n = 9, 81.8%) or an audio recording device (n = 3, 27.3%). In most cases, the patient (n = 6, 54.5%) or the accompanying person (n = 4, 36.4%) did the recording. In two cases (18.2%), the physicians themselves did the recordings and provided them to patients on a CD. Of all participants, 21 (22.3%) were aware of patients having secretly recorded a consultation (i.e., without the recording being provided to them), while 20 (21.3%) had an unconfirmed suspicion.

#### Desire for future consultation recordings

Participants were asked whether they would like to offer consultation recordings to patients in the future. Thirteen of the 94 participants (13.8%) answered “yes”, 29 (30.9%) answered “maybe”, and 52 (55.3%) answered “no”. Of those having answered “yes” or “maybe” (n = 42), 27 (64.3%) would be open to patients recording the consultation with their own cell phone, while 15 (35.7%) would not. Thirty-seven of those 42 participants (88.1%) thought that they should have access to the recording, while 5 (5.3%) did not. Ten of 40 participants (25.0%) would not listen to the recordings afterwards, while 13 (32.5%) would. Two participants did not answer this question. Seventeen participants (42.5%) said they would listen to the recording only in certain situations, for example if there were misunderstandings, conflicts, or further questions.

All participants were asked whether they would like to do consultation recordings if they or a relative were the patients. Twenty-one (22.3%) answered “yes”, 26 (27.7%) answered “maybe”, and 47 (50.0%) answered “no”.

## Discussion

Our initial exploration of the views on consultation recordings of physicians working in oncology in Germany revealed mixed attitudes. Yet, despite the mixed attitudes identified in this study, the rich dataset gathered offers insights on how to move along with this intervention in research and clinical practice. Participants acknowledged the potential benefits, particularly regarding information recall, comprehension, and sharing with relatives. However, they also strongly agreed with concerns associated with such recordings. Their negative views primarily revolved around misuse of the recordings. A minority of participants in our sample reported prior experiences and some had experienced or suspected that patients had covertly recorded medical encounters. Only a small percentage of participants expressed a definite willingness to offer consultation recordings to patients in the future, while a notable portion remained undecided.

In contrast to the predominantly positive attitudes found in our survey with cancer patients in Germany^24^, the physician sample demonstrated significant apprehension, particularly regarding recordings having the potential to negatively impact them on a personal level. A significant aspect of these concerns is losing control over the consultation process, which aligns with prior research^12,16,17^. Physicians may worry that after recordings are made, whether provided or made covertly, they will no longer have control over how patients handle or use them. Nevertheless, participants agreed that consultation recordings could be beneficial for patients. These findings are consistent with results from international research^2,6,12,16–20^.

Notably, our sample had limited experience with consultation recordings, which was less than reported in other studies (e.g. 28% of physicians^6^ and 93% of oncologists in the US^16^, 71% of physicians and nurses in the Netherlands^12^). This is in correspondence with our previous survey with patients with cancer that also found limited experience (i.e., about 5% reported having recorded medical encounters in the past)^24^. However, most of those physicians who reported prior experiences with consultation recordings, had recorded on multiple occasions. This could suggest that familiarity with consultation recordings may increase acceptance.

A considerable amount of participating physicians was (maybe) open to offer consultation recordings to patients in the future. Among those, there was a strong preference for maintaining control over the recording process, with most respondents desiring access to the recordings. This suggests that while there is an openness to consultation recordings, there is also a need for measures that ensure transparency and protect both patient and physician interests, which is also supported by prior research^12,16,17,19,26,38^. One proposed solution is for physicians to proactively offer patients to record consultations, thereby helping physicians maintain control while accommodating patient needs^26,39,40^. Moreover, encouraging patients to record consultations can be a sign of trust and openness and circumvents covert recordings^13,39,40^.

In terms of feasibility, our study findings suggest that participants do not consider the intervention to be a universally applicable solution for all patients. Instead, they believe it is suitable for specific groups of patients or in specific situations. They primarily identify benefits in two scenarios: (1) during discussions that involve many and complex information (for instance, consultations in which treatment decisions are made), and (2) for patients who struggle with recall and understanding. However, for routine implementation in healthcare, the perceived risks are viewed as excessive, with concerns that they may lead to more disadvantages than advantages, especially in relation to the physician–patient relationship.

As the use of digital technologies in healthcare continues to grow^41^, it is likely that consultation recordings will become more prevalent, requiring healthcare professionals to adapt^42^. However, the adoption of this practice in Germany seems to be at a relatively low level in international comparison^6,12,13,16^. This may be due to physicians in Germany being more skeptical to technological and digital innovations and shifts in power dynamics between physicians and patients^41^. It is also noteworthy that German law requires the consent of both parties for recording, unlike most US states and the UK, where one-party consent is sufficient^43^.

### Strengths and limitations

This is the first study to investigate the attitudes and experiences toward consultation recordings of oncologists in Germany, closing a relevant research gap. Another strength are the preceding qualitative interviews that informed the development of the quantitative questionnaire. However, this study faced several limitations, particularly regarding potential selection bias. Physicians who chose to respond to the survey might have had a more favorable attitude toward consultation recordings. Conversely, it is also possible that physicians opposed to the idea of consultation recordings were more eager to participate, possibly explaining our finding of less experience with such recordings compared to international research. In addition, data collection proved challenging. Despite employing multiple recruitment strategies, including personal outreach and social media, participation remained limited. Efforts to recruit through personal means, such as distributing leaflets, were often met with hesitation or reluctance to engage upon learning about its topic. Nevertheless, our findings align with prior studies, which also reported that approximately half of the participants held positive views on consultation recordings, suggesting consistent attitudes among physicians despite these recruitment challenges. Furthermore, the study focused on descriptive analyses, therefor no a priori power analyses were conducted, limiting the external validity of the results. Moreover, we cannot provide information on the number of individuals that were reached by our study invitations, as we used various recruitment strategies and asked for invitations to be forwarded. Therefore, we were not able to calculate the response rate. Additionally, we had no standardized and psychometrically tested questionnaire to assess the attitudes toward consultation recordings.

### Research and practice implications

The mixed attitudes of physicians found in this study seem to be a barrier to implementation of consultation recordings in Germany. Presenting evidence regarding benefits and concerns and fostering positive experiences with consultation recordings may help alleviate physicians concerns^12,16,17,26^, supporting a shift toward greater use of consultation recordings in Germany. Notably, a study involving more than 2700 video recordings provided to patients in a high-risk medical specialty, specifically a neurosurgical clinic, found no medico-legal issues^44^, offering valuable evidence that could increase acceptance among physicians. In order to gather further evidence and to assess the intervention’s feasibility within the German healthcare system, pilot testing in routine cancer care is needed. Furthermore, the next steps should involve adopting more robust study designs, which would include appropriate power analyses. In order to test generalizability, it should also be explored whether the results of this study can be reproduced in a representative sample and in other healthcare contexts and specialties.

### Conclusions

This was the first study to assess the attitudes and experiences of oncologists toward consultation recordings in Germany. Although potential benefits of this intervention were acknowledged, significant concerns were also highlighted. The limited experience with consultation recordings in our sample of oncologists indicated that the potential of this intervention is largely underutilized in German cancer care. Nonetheless, our study provided a foundation for further assessment and exploration of consultation recordings in Germany. Consultation recordings could become a valuable tool in routine cancer care, if current physician concerns were successfully overcome.

## Electronic supplementary material

Below is the link to the electronic supplementary material.

Supplementary Material 1

## Data Availability

The data supporting the findings of this study are available from the corresponding author on reasonable request.

## References

[1] Matsuyama, R. K., Kuhn, L. A., Molisani, A. & Wilson-Genderson, M. C. Cancer patients’ information needs the first nine months after diagnosis. *Patient Educ. Couns.* **90**, 96–102 (2013). 23058682 10.1016/j.pec.2012.09.009

[2] Tsulukidze, M., Durand, M. A., Barr, P. J., Mead, T. & Elwyn, G. Providing recording of clinical consultation to patients – a highly valued but underutilized intervention: A scoping review. *Patient Educ. Couns.* **95**, 297–304 (2014). 24630697 10.1016/j.pec.2014.02.007

[3] Kessels, R. P. C. Patients’ memory for medical information. *J. R. Soc. Med.* **96**, 219–222 (2003). 12724430 10.1258/jrsm.96.5.219 PMC539473

[4] van der Meulen, N., Jansen, J., van Dulmen, S., Bensing, J. & van Weert, J. Interventions to improve recall of medical information in cancer patients: A systematic review of the literature. *Psycho-Oncol.* **17**, 857–868 (2008). 10.1002/pon.1290 18050149

[5] Sherlock, A. & Brownie, S. Patients’ recollection and understanding of informed consent: A literature review. *ANZ J. Surg.* **84**, 207–210 (2014). 24812707 10.1111/ans.12555

[6] Barr, P. J. et al. Audio-/videorecording clinic visits for patient’s personal use in the United States: Cross-sectional survey. *J. Med. Internet Res.* **20**, e11308 (2018). 30209029 10.2196/11308 PMC6231772

[7] Rieger, K. L., Hack, T. F., Beaver, K. & Schofield, P. Should consultation recording use be a practice standard? A systematic review of the effectiveness and implementation of consultation recordings. *Psycho-Oncol.* **27**, 1121–1128 (2018). 10.1002/pon.4592 29178602

[8] Hyatt, A. et al. Culturally and linguistically diverse oncology patients’ perspectives of consultation audio-recordings and question prompt lists. *Psycho-Oncol.* **27**, 2180–2188 (2018). 10.1002/pon.4789 29893041

[9] Dommershuijsen, L. J., Dedding, C. W. M. & Van Bruchem-Visser, R. L. Consultation recording: What is the added value for patients aged 50 years and over? A systematic review. *Health Commun.* **36**, 168–178 (2019). 31556750 10.1080/10410236.2019.1669270

[10] Kwon, D. H., Aggarwal, R. R., Esserman, L. J. & Belkora, J. K. Prime time for consultation audio recordings: Supporting shared decision making during and after the COVID-19 era. *JCO Oncol. Pract.* **17**, 161–163 (2021). 33332174 10.1200/OP.20.00765

[11] Petric, J., Sadri, B., van Essen, P. & Dean, N. R. Improving preoperative breast reconstruction consultations: A qualitative study on the impact of personalised audio-recordings. *BMC Womens Health* **21**, 389 (2021). 34742266 10.1186/s12905-021-01534-8 PMC8571820

[12] van Bruinessen, I. R., Leegwater, B. & van Dulmen, S. When patients take the initiative to audio-record a clinical consultation. *Patient Educ. Couns.* **100**, 1552–1557 (2017). 28302340 10.1016/j.pec.2017.03.001

[13] Elwyn, G., Barr, P. J. & Grande, S. W. Patients recording clinical encounters: a path to empowerment? Assessment by mixed methods. *BMJ Open* **5**, e008566 (2015). 26264274 10.1136/bmjopen-2015-008566 PMC4538278

[14] Hyatt, A. et al. Testing consultation recordings in a clinical setting with the secondears smartphone app: Mixed methods implementation study. *JMIR Mhealth Uhealth* **8**, e15593 (2020). 31961333 10.2196/15593 PMC7001044

[15] Smith, S. M., Stelmar, J., Lee, G., Carroll, P. R. & Garcia, M. G. Use of voice recordings in the consultation of patients seeking genital gender-affirming surgery: An opportunity for broader application throughout surgery?. *J. Surg. Res. (Houst)* **5**, 618–625 (2022). 36643404 10.26502/jsr.10020269 PMC9836232

[16] Jimenez, R. B. et al. Do you mind if I record?: Perceptions and practice regarding patient requests to record clinic visits in oncology. *Cancer* **128**, 275–283 (2022). 34633655 10.1002/cncr.33910

[17] Ryan, L., Weir, K., Maskell, J. & Le Brocque, R. Smartphone standoff: A qualitative study exploring clinician responses when a patient uses a smartphone to record a hospital clinical encounter. *BMJ Open* **12**, e056214 (2022). 35459670 10.1136/bmjopen-2021-056214 PMC9036419

[18] Joshi, A., Farberov, M., Demissie, S., Smith, M. C. & Elwyn, G. Attitudes of physicians to recording clinical encounters: Responses to an online survey. *J. Gen. Intern. Med.* **35**, 942–943 (2020). 31270783 10.1007/s11606-019-05127-y PMC7080903

[19] Moloczij, N. et al. Barriers and facilitators to the implementation of audio-recordings and question prompt lists in cancer care consultations: A qualitative study. *Patient Educ. Couns.* **100**, 1083–1091 (2017). 28117193 10.1016/j.pec.2017.01.005

[20] Ivermee, C. & Yentis, S. M. Attitudes of postnatal women and maternity staff towards audio recording of consent discussions. *Anaesthesia* **74**, 1095–1100 (2019). 30973191 10.1111/anae.14660

[21] Hack, T. F., Ruether, J. D., Weir, L. M., Grenier, D. & Degner, L. F. Promoting consultation recording practice in oncology: Identification of critical implementation factors and determination of patient benefit. *Psychooncology* **22**, 1273–1282 (2013). 22821445 10.1002/pon.3135

[22] Pringle, M. Does awareness of being video recorded affect doctors’ consultation behaviour?. *Br. J. Gen. Pract.* **40**, 455–458 (1990). 2271278 PMC1371415

[23] Prictor, M., Johnston, C. & Hyatt, A. Overt and covert recordings of health care consultations in Australia: Some legal considerations. *Med. J. Aust.* **214**, 119 (2021). 33131072 10.5694/mja2.50838

[24] Topf, C., Scholl, I. & Hahlweg, P. Attitudes and experiences of cancer patients toward the provision of audio recordings of their own medical encounter: A cross-sectional online survey. *Front. Psychol.*10.3389/fpsyg.2024.1378854 (2024). 38962233 10.3389/fpsyg.2024.1378854 PMC11220273

[25] Hack, T. F. et al. Impact of consultation recordings on patient-reported outcomes in patients with brain tumors: A parallel randomized controlled trial. *Support. Care Cancer* **29**, 5681–5690 (2021). 33595717 10.1007/s00520-021-06038-7

[26] Ryan, L., Weir, K. A., Maskell, J., Bevan, L. & Le Brocque, R. Beyond recording the clinical discussion: A qualitative study into patient-led recordings in hospital. *J. Patient Exp.*10.1177/23743735231203126 (2023). 37781068 10.1177/23743735231203126 PMC10540596

[27] Damschroder, L. J., Reardon, C. M., Widerquist, M. A. O. & Lowery, J. The updated consolidated framework for implementation research based on user feedback. *Implement. Sci.*10.1186/s13012-022-01245-0 (2022). 36309746 10.1186/s13012-022-01245-0 PMC9617234

[28] Eysenbach, G. Improving the quality of web surveys: The checklist for reporting results of internet E-surveys (CHERRIES). *J. Med. Internet Res.* **6**, e34 (2004). 15471760 10.2196/jmir.6.3.e34 PMC1550605

[29] Mayring, P. & Fenzl, T. Qualitative Inhaltsanalyse. In *Handbuch Methoden der empirischen Sozialforschung* 633–648 (Springer Fachmedien Wiesbaden, Wiesbaden, 2019). 10.1007/978-3-658-21308-4_42.

[30] Scholl, I., Zill, J. M., Härter, M. & Dirmaier, J. An integrative model of patient-centeredness—A systematic review and concept analysis. *PLoS ONE* **9**, e107828 (2014). 25229640 10.1371/journal.pone.0107828 PMC4168256

[31] Lipson-Smith, R. et al. Are audio recordings the answer?—A pilot study of a communication intervention for non-English speaking patients with cancer. *Psycho-Oncol.* **25**, 1237–1240 (2016). 10.1002/pon.4193 27291636

[32] Elwyn, G., Barr, P. J. & Castaldo, M. Can patients make recordings of medical encounters?. *JAMA* **318**, 513–514 (2017). 28692707 10.1001/jama.2017.7511

[33] Degner, L. F., Sloan, J. A. & Venkatesh, P. The control preferences scale. *Can. J. Nurs. Res.* **29**, 21–43 (1997). 9505581

[34] Wessel, D., Attig, C. & Franke, T. ATI-S - An ultra-short scale for assessing affinity for technology interaction in user studies. In *Proceedings of Mensch und Computer 2019* 147–154 (ACM, New York, NY, USA, 2019). 10.1145/3340764.3340766.

[35] Ward, M. K. & Meade, A. W. Dealing with careless responding in survey data: prevention, identification, and recommended best practices. *Annu. Rev. Psychol.* **74**, 577–596 (2023). 35973734 10.1146/annurev-psych-040422-045007

[36] Sheskin, D. J. *Handbook of Parametric and Nonparametric Statistical Procedures* (Chapman and Hall/CRC, 2011).

[37] Franke, T., Attig, C. & Wessel, D. A Personal resource for technology interaction: Development and validation of the affinity for technology interaction (ATI) scale. *Int. J. Hum.-Comput. Interact.* **35**, 456–467 (2019).

[38] Ryan, L., Weir, K. A., Maskell, J., Bevan, L. & Le Brocque, R. ‘What are you hiding from me?’ A qualitative study exploring health consumer attitudes and experiences regarding the patient-led recording of a hospital clinical encounter. *Health Expect.* **25**, 3096–3104 (2022). 36229999 10.1111/hex.13617 PMC9700167

[39] Tsulukidze, M., Grande, S. W., Thompson, R., Rudd, K. & Elwyn, G. Patients covertly recording clinical encounters: Threat or opportunity? A qualitative analysis of online texts. *PLoS ONE* **10**, e0125824 (2015). 25933002 10.1371/journal.pone.0125824 PMC4416897

[40] Elwyn, G. & Buckman, L. Should doctors encourage patients to record consultations?. *BMJ* **350**, g7645 (2015). 25569249 10.1136/bmj.g7645

[41] Hansen, A. et al. Perception of the progressing digitization and transformation of the German health care system among experts and the public: Mixed methods study. *JMIR Public Health Surveill.* **5**, e14689 (2019). 31661082 10.2196/14689 PMC6913772

[42] Rodriguez, M., Morrow, J. & Seifi, A. Ethical implications of patients and families secretly recording conversations with physicians. *JAMA* **313**, 1615–1616 (2015). 25763514 10.1001/jama.2015.2424

[43] Elwyn, G., Engel, J., Scalia, P. & Shachar, C. Individuals recording clinical encounters. *Commun. Med.* **19**, 58–76 (2024). 39916094 10.1558/cam.20257

[44] Meeusen, A. J. & Porter, R. Patient-reported use of personalized video recordings to improve neurosurgical patient-provider communication. *Cureus*10.7759/cureus.273 (2015). 26180697 10.7759/cureus.273 PMC4494565

