# Supplementary material for: A cross-sectional online survey on oncologists’ attitudes toward and experiences with providing patients with audio recordings of their medical encounters
Source: Sci Rep. 2025 May 16;15:17100. doi: 10.1038/s41598-025-01962-8 (PMC12084334; doi:10.1038/s41598-025-01962-8)
Supplement: Supplementary file 1 — Supplementary Material 1 [file 41598_2025_1962_MOESM1_ESM.pdf]

SUPPLEMENTARY FILE 1

"Improving the Quality of Web Surveys: The Checklist for Reporting Results of Internet E-Surveys (CHERRIES") [1]

Item Category	Checklist Item	Explanation	Answer
**Design**	Describe survey design	Describe target population, sample frame. Is the sample a convenience sample? (In "open" surveys this is most likely.)	See page 2.
**IRB (Institutio-nal Review Board) appro-val and infor-med consent process**	IRB approval	Mention whether the study has been approved by an IRB.	See page 9.
	Informed consent	Describe the informed consent process. Where were the participants told the length of time of the survey, which data were stored and where and for how long, who the investigator was, and the purpose of the study? process	See page 9. Participants were informed about duration of the online survey, data processing and safekeeping, study purpose and investigators, prior to participating in the online survey.
	Data protection	If any personal information was collected or stored, describe what mechanisms were used to protect unauthorized access.	A detailed data protection concept was developed prior to the beginning of data collection.
**Development and pre-testing**	Development and testing	State how the survey was developed, including whether the usability and technical functionality of the electronic questionnaire had been tested before fielding the questionnaire.	See page 3.
**Recruitment process and description of the sample having access to the questionnaire**	Open survey versus closed survey	An "open survey" is a survey open for each visitor of a site, while a closed survey is only open to a sample which the investigator knows (password-protected survey).	See page 4.
	Contact mode	Indicate whether or not the initial contact with the potential participants was made on the Internet. (Investigators may also send out questionnaires by mail and allow for Web-based data entry.)	See page 4.
	Advertising the survey	How/where was the survey announced or advertised? Some examples are offline media (newspapers), or online (mailing lists – If yes, which ones?) or banner ads (Where were these banner ads posted and what did they look like?). It is important to know the wording of the announcement as it will heavily influence who chooses to participate. Ideally the survey announcement should be published as an appendix.	See page 4. Wordings of the survey announcement can be requested via E-Mail.

**Survey administration**	Web/E-Mail	State the type of e-survey (eg, one posted on a Web site, or one sent out through e-mail). If it is an e-mail survey, were the responses entered manually into a database, or was there an automatic method for capturing responses?	See page 4. Data was automatically captured through LimeSurvey[2].
	Context	Describe the Web site (for mailing list/newsgroup) in which the survey was posted. What is the Web site about, who is visiting it, what are visitors normally looking for? Discuss to what degree the content of the Web site could pre-select the sample or influence the results. For example, a survey about vaccination on an anti-immunization Web site will have different results from a Web survey conducted on a government Web site	Does not apply. / See page 4.
	Mandatory/voluntary	Was it a mandatory survey to be filled in by every visitor who wanted to enter the Web site, or was it a voluntary survey?	Does not apply.
	Incentives	Were any incentives offered (eg, monetary, prizes, or non-monetary incentives such as an offer to provide the survey results)?	See page 4.
	Time/Date	In what timeframe were the data collected?	See page 4. The survey was online from June 2022 to April 2023 and October 2023 to April 2024.
	Randomization of items or questionnaires	To prevent biases items can be randomized or alternated.	There was no randomization of items.
	Adaptive questioning	Use adaptive questioning (certain items, or only conditionally displayed based on responses to other items) to reduce number and complexity of the questions.	There several adaptive questions; see page 3 and the Supplementary file 3.
	Number of Items	What was the number of questionnaire items per page? The number of items is an important factor for the completion rate.	There was a total of 80 questions distributed unevenly on 10 screens.
	Number of screens (pages)	Over how many pages was the questionnaire distributed? The number of items is an important factor for the completion rate.	There were 10 screens in total, of which 7 included survey items and 3 included the start page, study information and checking of inclusion criteria.
	Completeness check	It is technically possible to do consistency or completeness checks before the questionnaire is submitted. Was this done, and if “yes”, how (usually JAVAScript)? An alternative is to check for completeness after the questionnaire has been submitted (and highlight mandatory items). If this has been done, it should be reported. All items should provide a non-response option such as “not applicable” or “rather not say”, and selection of one response option should be enforced.	Every question was mandatory; they were “no response” options provided in some cases See Supplementary file 3.

	Review step	State whether respondents were able to review and change their answers (eg, through a Back button or a Review step which displays a summary of the responses and asks the respondents if they are correct).	No, respondents were not able to change their answers.
**Response rates**	Unique site visitor	If you provide view rates or participation rates, you need to define how you determined a unique visitor. There are different techniques available, based on IP addresses or cookies or both.	No measures were taken in order to prevent multiple entries from the same individual, in order to ensure maximal anonymity of participants.
	View rate (Ratio of unique survey visitors/unique site visitors	Requires counting unique visitors to the first page of the survey, divided by the number of unique site visitors (not page views!). It is not unusual to have view rates of less than 0.1 % if the survey is voluntary.	Does not apply.
	Participation rate (Ratio of visitors who agreed to participate)/unique first survey page visitors	Count the unique number of people who filled in the first survey page (or agreed to participate, for example by checking a checkbox), divided by visitors who visit the first page of the survey (or the informed consents page, if present). This can also be called “recruitment” rate.	128:178=0,72
	Completion rate (Ratio of users who finished the the survey/users who agreed to participate	The number of people submitting the last questionnaire page, divided by the number of people who agreed to participate (or submitted the first survey page). This is only relevant if there is a separate “informed consent” page or if the survey goes over several pages. This is a measure for attrition. Note that “completion” can involve leaving questionnaire items blank. This is not a measure for how completely questionnaires were filled in. (If you need a measure for this, use the word “completeness rate”.)	94:128=0,73<br>(94 participants met the inclusion criteria)
**Preventing multiple entries from the same individual**	Cookies used	Indicate whether cookies were used to assign a unique user identifier to each client computer. If so, mention the page on which the cookie was set and read, and how long the cookie was valid. Were duplicate entries avoided by preventing users access to the survey twice; or were duplicate database entries having the same user ID eliminated before analysis? In the latter case, which entries were kept for analysis (eg, the first entry or the most recent)?	No measures were taken in order to prevent multiple entries from the same individual, in order to ensure maximal anonymity of participants.

	IP check	Indicate whether the IP address of the client computer was used to identify potential duplicate entries from the same user. If so, mention the period of time for which no two entries from the same IP address were allowed (eg, 24 hours). Were duplicate entries avoided by preventing users with the same IP address access to the survey twice; or were duplicate database entries having the same IP address within a given period of time eliminated before analysis? If the latter, which entries were kept for analysis (eg, the first entry or the most recent)?	No measures were taken in order to prevent multiple entries from the same individual, in order to ensure maximal anonymity of participants.
	Log file analysis	Indicate whether other techniques to analyze the log file for identification of multiple entries were used. If so, please describe.	No measures were taken in order to prevent multiple entries from the same individual, in order to ensure maximal anonymity of participants.
	Registration	In “closed” (non-open) surveys, users need to login first and it is easier to prevent duplicate entries from the same user. Describe how this was done. For example, was the survey never displayed a second time once the user had filled it in, or was the username stored together with the survey results and later eliminated? If the latter, which entries were kept for analysis (eg, the first entry or the most recent)?	Does not apply.
**Analysis**	Handling of incomplete questionnaires	Were only completed questionnaires analyzed? Were questionnaires which terminated early (where, for example, users did not go through all questionnaire pages) also analyzed?	See page 4.
	Questionnaire submitted with an atypical timestamp	Some investigators may measure the time people needed to fill in a questionnaire and exclude questionnaires that were submitted too soon. Specify the timeframe that was used as a cut-off point, and describe how this point was determined.	The lowest time to completion was about 3 minutes. This can be considered a realistic time to completion.
	Statistical correction	Indicate whether any methods such as weighting of items or propensity scores have been used to adjust for the non-representative sample; if so, please describe the methods.	No measures in this form were taken.

References

1. Eysenbach G. Improving the quality of Web surveys: the Checklist for Reporting Results of Internet E-Surveys (CHERRIES). J Med Internet Res 2004; 6(3):e34.

2. Limesurvey GmbH. / LimeSurvey: An Open Source survey tool /LimeSurvey GmbH, Hamburg, Germany. URL http://www.limesurvey.org.

SUPPLEMENTARY FILE 2

Qualitative Semi-Structured Interviews: Methods, Results, and Additional Documents

1 Methods

1.1 Participants

Oncologists were eligible to participate. It was planned to include 10 participants in the qualitative interviews.

1.2 Materials and questionnaires

The interviews followed an interview guideline that we developed for this purpose (cp. **3.1 Interview Guide)**. The guideline included questions about participants' experiences with consultation recordings and their attitudes towards it. An additional anonymous questionnaire, provided to participants before the interview, included demographic questions (e.g. age, gender, professional experience) (cp. **3.2 Demographic Questionnaire**).

1.3 Data collection

Participants were recruited using convenience sampling. They were invited via email through collaboration partners. Participants received a consent form and the demographic questionnaire prior to participation. The interviews were conducted only after they had provided both written and verbal informed consent. Due to restrictions during the Covid-19 pandemic, interviews were held via telephone. The interviews were conducted between February 2022 and March 2022. Participants could receive an incentive of 25 Euros.

1.4 Data analysis

The telephone interviews were audio-recorded, transcribed by an external contractor, and checked and anonymized by the study team. FS, AS (cp. **Acknowledgements**), and CT analyzed the data using qualitative content analysis (Mayring & Fenzl, 2019), using the software MaxQDA (version 20). One of the PIs of the study (PH) repeatedly provided feedback during the analysis. The data was analyzed inductively, with the selection criteria being 1) concrete statements regarding participants' experiences

with consultation recordings or 2) concrete statements regarding participants' attitudes towards consultation recordings (cp. **3.3 Evaluation Plan**). Descriptive statistics of the demographic data were analyzed using SPSS 27.

2 Results

10 participants were interviewed. Participant characteristics are presented in **Table 1.** The interviews were between 27 and 60 minutes (*mean*=39 minutes, SD=11 minutes).

Table 1

Participant characteristics (N=10)

	Mean	SD	Min	Max
**Age in years**	45	15	28	69
	N		%	
**Sex**				
Female	2		20	
Male	8		80	
**Education level**[1]				
In residency training	3		30	
Completed residency	7		70	
**Current position / work setting**				
Junior physician at a hospital	4		40	
Senior or head physician at a hospital	2		20	
Working in own outpatient practice	1		10	
Employed in an outpatient practice	3		30	
**Experience in oncology**	.			
Less than 5 years	4		40	
5-10 years	1		10	
11-20 years	2		20	
More than 20 years	3		30	

Interview participants were found to have some prior experiences with audio recordings. When asked about their attitudes, we found a wide range of aspects that participants expected to be influenced by consultation recordings. The complete coding tree is presented in **Table 2**.

Table 2

Codes and code descriptions

ID	Codes
**E**	**Research Question 1 - Experiences**
**E1**	**No experiences**
**E2**	**Similar experiences**
E2.1	Family members participate via phone
E2.2	Recording of consultations for training purposes
E2.3	Live transmission of consultations for educational purposes
**E3**	**Experiences**
E3.1	Few to frequent experiences
E3.2	Patients made recordings without asking for permission
E3.3	Patients' request for recording was rejected
**A**	**Research Question 2 - Attitudes**
**A1**	**Expected changes on the part of the patient**
A1.1	Enhanced information transmission, processing, and reflection
A1.2	Facilitation of feeling secure and informed
A1.3	Promotion of shared-decision making
A1.4	Helpful for empowered management of the disease by the patient
A1.5	Emotional-therapeutic benefit (including coping with the disease)
A1.6	Concerns about patients being overwhelmed and unsettled by listening
A1.7	Concerns about excessive occupation with the disease
A1.8	Second opinions
A1.8.1	*Facilitates obtaining second opinions*
A1.8.2	*Concerns about obtaining second opinions*
A1.9	No impact on being well informed
A1.10	No impact on shared decision-making
A1.11	No benefit for empowerment
**A2**	**Physician-patient communication**
A2.1	More precise/appropriate/understandable communication

**A3**	**Physician-patient relationship**
A3.1	Burden on the relationship
A3.2	Relationship-building
A3.3	Demonstration of openness/transparency
A3.4	No impact on the relationship
**A4**	**Patient safety**
A4.1	Useful as legal protection
A4.2	Facilitation of patient adherence
A4.3	Evidence of malpractice
A4.4	Facilitation of safe treatment (e.g., regarding medication)
A4.5	Facilitation of comparison between physicians
A4.6	No impact on patient safety
**A5**	**Expected changes on the part of the physician**
A5.1	Insecurity and behavioral change due to recording situation
A5.2	Benefits for physicians' information gathering
A5.3	Helpful for improvement in communication/training purposes
A5.4	No insecurity due to adaptation to the recording situation
A5.5	No benefit from consultation recordings for physicians
**A6**	**Involvement of family members**
A6.1	Sharing as support and relief for patients
A6.2	Concerns about certain topics
A6.3	Sharing as a burden for patients
A6.4	Pressure on physicians through family members
**A7**	**Practical-technical implementation**
A7.1	Flexible recording and access modes
A7.2	Increased effort due to consultation recording (time-related, organizational)
A7.3	Consultation recordings should be isolated cases
A7.4	Concerns about technical implementation
A7.5	Only partial recording of the consultation
A7.6	Feasibility depends on the setting

A7.7	Reduced time expenditure due to conversation recording
A7.8	Inform patients about consultation recordings before appointment
A7.9	Consultation recordings should be routine practice
A7.10	Live transmission of consultation instead of recording
A7.11	Voluntariness on the patient side is essential
**A8**	**Collaboration between physicians/healthcare professionals**
A8.1	Benefit for other healthcare professionals
A8.2	Concerns about sharing with other healthcare professionals
A8.3	Listen is too time-consuming for other healthcare professionals
A8.4	No or minimal changes in collaboration
**A9**	**Different groups of people**
A9.1	Particularly helpful for certain groups of people
A9.2	Concerns with certain groups of people
A9.3	Helpful for all kind of people
**A10**	**Different consultation occasions/types**
A10.1	Helpful for emotional conversations (initial diagnosis/palliative situation)
A10.2	Helpful for therapy planning and pre-treatment consultation
A10.3	Consultation recordings not useful for every consultation
A10.4	Helpful for all consultation types
**A11**	**General conditions in Germany**
A11.1	Concerns about (legal) consequences
A11.2	Concerns about data protection
A11.3	Insufficiently established

3 Additional Documents

3.1 Interview Guide

Preparation checklist for interviewer

- Informed consent given?
- Demographic questionnaire received?
- Administrative form for incentives received?
- Two recording devices prepared?

Interview

Introduction

- *The interviewer (CT) calls the participant at the agreed-upon time.*
- "Hello [Mrs./Mr./Dr. X], my name is Cheyenne Topf. As you know, I am calling for our scheduled telephone interview. Thank you once again for agreeing to participate. The interview will take approximately 30-60 minutes. I have received your consent form, the incentives form, and the short questionnaire. Do you have any further questions? If not, we can proceed with the interview, and as discussed, I will now start the audio recording."
- *Turn on the audio recording device*
- "First, I need the anonymous code that you provided on the questionnaire. If you do not have it at hand, please answer the following questions:

1. What is the first letter of your mother's first name?
2. What is the first letter of your father's first name?
3. What is the last digit of your house number?
4. What is the last digit of your telephone or mobile number?"

- "As you are aware, these interviews are being conducted to gain insight into your perspective as an oncologist about providing an audio recording of your medical encounter to patients."
- "I will now ask you some questions one by one, and I kindly ask you to share everything that comes to your mind. For this interview, it is important that you feel comfortable expressing your opinions and experiences openly and honestly. There are no right or wrong answers. Feel free to share both critical and positive impressions."

Context:

- "The opportunity to record medical encounters and provide them to patients is more common in other countries such as the US, UK, and Australia than in Germany. This is why we are conducting this project in Germany."

Guiding Questions:

1. “When you hear about this, what is your initial impression of providing audio recordings of your medical encounters to patients?”

- “Next, I would like to discuss your ideas on how to implement such a practice.”

2. “How could these audio recordings be made and provided?”
   a. “Who could benefit from such recordings?”
   b. “In what situations could such recordings be helpful?”
   c. “Why do you think would patients listen to the audio recordings afterward?”
   d. “When and with whom do you think would patients listen to the audio recordings afterward?”
   e. “Would you like to have access to the audio recordings as well?”
      i. *If yes:* “Why?”
3. “How would you respond to a patient’s request to record the medical encounter?”

- *If the participant hints at previous experiences:*

4. “What experiences have you had with providing an audio recording of your medical encounters to patients?”

- “I would now like you to imagine being in a medical encounter and providing such an audio recording to a patient. You can think about past or future encounters.”

5. “What could be an advantage or helpful about recording medical encounters?”

- *Follow up until no more points are mentioned; if the participant shares experiences, ask about what else they can think of.*

6. “What might be challenging or disadvantageous about making such recordings?”

- *Follow up until no more points are mentioned; if the participant shares experiences, ask about what else they can think of.*

7. “What changes would you expect if you were providing recordings of your encounters to your patients?”
   a. “How could the recording change how well-informed patients are and how much they know about their condition and treatment?” (Dimension: Patient information)

b. “How could the recording change patients’ involvement in treatment decisions?” (Dimension: Patient involvement)
c. “How could the recording change how actively and independently patients manage their condition and treatment?” (Dimension: Empowerment)
d. “How could the recording change the relationship between you and your patients?” (Dimension: Provider-patient relationship)
e. “How could the recording change communication with your patients?” (Dimension: Provider-patient communication)
f. “How could the recording change the involvement of your patients’ family members?” (Dimension: Involvement of family members)
g. “How could the recording change collaboration among different healthcare providers?” (Dimension: Coordination and continuity; integration of medical and non-medical care)
h. “How could the recording affect the patient safety?” (Dimension: Patient safety)

- “Now that we have discussed your thoughts on such recordings, I would like to ask a few questions about your personal competency with digital devices and in legal matters.”

8. “How confident do you feel in using digital devices such as smartphones, computers, or tablets?”
    a. “What is your view on communicating with patients over the Internet? For example, via email or digitally communicating test results?”

- *Follow up if not mentioned*
    b. “Do you know how to send and receive emails?”
    c. “Do you know how to make an audio recording?”
    d. “Do you know how to connect a USB drive to a computer and use the data on it?”

9. “How confident do you feel in handling legal matters, for example regarding data protection or medical liability issues?”

- “Finally, I would like to ask you:”

10. “How would you respond to a patient’s request to record the medical encounter **now**? Has anything changed about this since the beginning of the interview?”

Summary:

11. “When you think back to everything we have discussed today; what aspects are particularly important to you?”
12. “Is there anything we haven't discussed that must not be overlooked?”

Conclusion:

- *Thank you and goodbye*
- *Turn off the audio recording device*

3.2 Demographic Questionnaire

Dear participant,

With this questionnaire, we would like to gather some anonymous information about you and your experience in treating patients with cancer. Most questions can be answered by checking the appropriate boxes. For other questions, you can enter your response in the open text fields. It should take approximately 5 minutes to complete the questionnaire.

All information provided is confidential, and all project team members are bound by confidentiality agreements. In order to link the information from the questionnaire to the telephone interview, we kindly ask you to provide a code below. We will ask for this code again at the beginning of the interview but will not associate it with your identity. The evaluation will be conducted anonymously at the Institute for Medical Psychology at the University Medical Center Hamburg-Eppendorf. We are very grateful for your participation in this project and thank you warmly for your support.

To create an anonymous code for you, we need the following information:

- First letter of your mother's first name:
- First letter of your father's first name:
- Last digit of your house number:
- Last digit of your telephone or mobile number:

Today's Date:
DD MM YYYY

Personal Information
Please provide some information about yourself:

1. Age: ___________________ (in years)
2. Gender:

   ☐ Female ☐ Male ☐ Non-binary/Other ☐ Prefer not to say
3. Education level:

   ☐ In residency training

   ☐ Completed residency

4. Current position / work setting:

   ☐ Junior physician at a hospital

   ☐ Senior or head physician at a hospital

   ☐ Working in own outpatient practice

   ☐ Employed in an outpatient practice

☐ other (please specify):________________________________

5. Experience in oncology:

   ☐ Less than 5 years

   ☐ 5-10 years

   ☐ 11-20 years

   ☐ More than 20 years

Thank you for completing the questionnaire!

3.3 Evaluation plan

Qualitative Content Analysis according to Mayring & Frenzl (2019)

Planned on: 07 September 2022, iteratively revised

Planned by: Funda Sahbudak, Alena Schneider, Cheyenne Topf, Pola Hahlweg

Material Definition: Transcripts of audio recordings of 10 interviews with 10 oncologists conducted as part of the study "Patient-centered cancer care by providing patients with audio recordings of medical encounters - a feasibility study."

Research Questions (RQ):

1) What experiences do oncologists report regarding consultation recordings?
2) What justifies the attitude of oncologists towards consultation recordings?

Analytical Approach (Inductive or Deductive): Inductive

Selection Criteria (Precise Definition):

1) Concrete statements regarding the experiences of a participants (PT) concerning consultation recordings. This includes coding statements when a PT reports having no experiences. "Experiences" here refer to knowledge gained through one's own perception and experience of consultation recordings (see https://dorsch.hogrefe.com/stichwort/erfahrung for reference). This may include descriptions of one's own behavior, thoughts, or feelings and may also involve the behavior of others.
2) Concrete statements explaining the attitude of a participant (PT) toward consultation recordings. "Attitude" here refers to the psychological disposition towards consultation recordings, associated with an judgement or expectation (see https://dorsch.hogrefe.com/stichwort/einstellung#search=cfe219c9c04e1f0f5f9d2c271735c848&offset=0). This can be, for example, positive, neutral, negative, undecided, or skeptical. The reasons provided for these specific attitudes will be coded. Additionally, each code will be assigned a direction to indicate whether the reason tends towards a positive or negative attitude.

Level of Abstraction: Concrete statements related to the selection criteria

Analysis Units:

- Coding unit (minimum): meaningful phrase
- Context unit (maximum): entire text section on a topic

- Evaluation unit: Entire transcript
- Multiple coding for different aspects possible
- Multiple coding within one transcript possible

Additional Note: If no experiences are reported throughout the entire transcript, the entire transcript will be coded with the label "no experiences."

Coding steps: Analysis will be conducted in successive stages for each research question.

A) FS and AS read through all the data material to get an overall impression.

B) Oncologist Experiences (RQ1)

1. AS codes 10% of the oncologist material (=1 transcript), in alignment with the code system of the study with cancer patients (see Supplementary file 2, Topf et al. 2024)
2. Joint discussion within the project team with AS, FS, CT, and PH.
3. AS codes the remaining oncologist material
4. Joint discussion within the project team with AS, FS, and PH.
5. Thorough review of the code labels for clarity and making any necessary adjustments by AS
6. FS codes the entire oncologist material (double coding)
7. Round of feedback and consensus discussion between AS and FS and making any necessary adjustments.
8. Evaluation validation by CT and further discussion within the team (CT, FS, AS) – for handover
9. Thorough review of codes and coded passages with revisions by CT
10. PH checks the code system and has a joint discussion with CT
11. CT incorporates feedback and finalizes the analysis for RQ 1

C) Oncologist Attitudes (RQ2)

1. AS and FS code 20% of the oncologist material (= 2 transcripts, double coding of the same material)
2. FS and AS discuss and compare their respective codings of the first 20% of the data material and the resulting codes, making necessary revisions.
3. Joint discussion within the project team with PH and further adjustment of the code system and codings.
4. AS codes the remaining oncologist material

5. FS reviews the codings and the code system for comprehensibility and consistency and adds if necessary.
6. Another round of feedback and consensus discussion between FS and AS, as well as adjustments to the code system and codings.
7. Evaluation validation by CT and further discussion within the team (CT, FS, AS) – for handover
8. CT checks the codings and the code system (including memos) for comprehensibility and consistency and conducts comprehensive revisions.
9. PH checks the code system and has a joint discussion with CT
10. CT incorporates feedback and finalizes the analysis for RQ 2

References

1. Mayring, P., & Fenzl, T. (2019). Qualitative Inhaltsanalyse. In *Handbuch Methoden der empirischen Sozialforschung* (pp. 633–648). Springer Fachmedien Wiesbaden. https://doi.org/10.1007/978-3-658-21308-4_42

SUPPLEMENTARY FILE 3

Quantitative online survey

Today's date						
	D	D	M	M	Y	Y

The provision of audio recordings of medical encounters for patients

Information and Consent Form for Participation in an Online Survey.

Dear Participants,

We would like to understand your thoughts on providing audio recordings of their own medical encounters to patients with cancer. We are particularly interested in the perspectives of patients with cancer and oncologists. Your participation is crucial to this project.

We kindly ask you to complete the following anonymous questionnaire. It includes questions about your experiences and attitudes, as well as some questions about yourself (e.g., age, gender, professional experience).

Filling out the questionnaire will take approximately 20 minutes. As a token of appreciation for your participation, you can receive a compensation of € 10 via bank transfer or a voucher (more information: www.wunschgutschein.de). The personal information required for this will be stored separately from your questionnaire and cannot be linked to your responses. Providing this information is voluntary.

Your participation is voluntary. Your data will only be used if you consent to participate. If you do not wish to participate, you will not face any disadvantages. You can withdraw at any time, even after giving consent and without providing reasons, by simply closing the window.

For the sake of readability, in some places, we have omitted the simultaneous use of male, female, and diverse gender forms (m/w/d). All references to individuals apply equally to all genders.

Project team:

Cheyenne Topf (Research Associate), Dr. Pola Hahlweg (Principal Investigator), Prof. Dr. Isabelle Scholl (Principal Investigator)

If you have read and understood the project and data protection information and wish to participate in the survey, please consent below. Please save this PDF so you can refer to your consent at any time. If you do not wish to participate, you do not need to take any action.

1. **Do you treat oncological patients?**

☐ Yes ☐ No

Experiences with the provision of audio recordings of medical encounters

The following pages inquire about your experiences regarding the provision of audio recordings of medical encounters.

2. **Have you ever had the experience of audio recording medical encounters?**

☐ Yes ☐ No

3. **How frequently have you made audio recordings of medical encounters?**

☐ Once ☐ Multiple times

4. *[If experience: „yes“:]* **Who suggested making the audio recording? (Multiple answers possible)**

☐ Myself ☐ My patient ☐ Accompanying person of my patient ☐ Another person *(please specify)*: ______________________________

5. *[If experience: „yes":]* **How was the audio recording made? (Multiple answers possible)**
   ☐ With an audio recording device ☐ With a cell phone ☐ No response ☐ Other method *(please specify)*: ______________________________

6. *[If experience: „yes":]* **Who made the audio recording? (Multiple answers possible)**
   ☐ Myseld ☐ My patient ☐ Accompanying person of my patients ☐ Another person *(please specify)*: ________________________

7. *[If audio recording made by physician:]* **How was the audio recording provided to the patient? (Multiple answers possible)**
   ☐ With a USB stick ☐ via Email ☐ over the internet (e.g. patient portal, cloud storage) ☐ anderer Weg *(bitte angeben)*: ________________________

8. *[If experience: „yes":]* **Was the audio recording also available to you?**
   ☐ Yes ☐ No

9. *[If available: „yes":]* **Did you listen tot he audio recording(s) again afterwards?**
   ☐ Yes ☐ No

10. **Have you ever noticed that patient secretly recorded a medical encounter?**
    ☐ Yes ☐ No ☐ Unconfirmed suspicion ☐ No response

Attitudes towards the provision of audio recordings of medical encounters

The following questions concern your attitudes towards providing audio recordings of medical encounters. Please answer based on your current feelings. There are no right or wrong answers. Please try to provide your level of agreement spontaneously and generally.

11. **In principle, it would be okay for me to offer a consultation recording to patients.**
    ☐ Does not apply at all ☐ Mostly does not apply ☐ Somewhat does not apply ☐ Somewhat applies ☐ Mostly applies ☐ Fully applies ☐No response

12. **I would allow patients to make a consultation recording, but I would not proactively offer it.**
    ☐ Does not apply at all ☐ Mostly does not apply ☐ Somewhat does not apply ☐ Somewhat applies ☐ Mostly applies ☐ Fully applies ☐No response

13. **How would you describe your attitude towards the provision of consultation recordings for patients?**
    ☐ Very negative ☐ Mostly negative ☐ Somewhat negative ☐ Somewhat positive ☐ Mostly positive ☐ Very positive ☐No response

Please check the extent to which you agree with the following statements. When the term „consultation recording" is used, it refers to the audio recording of medical encounters.

	Completely disagree	Partially disagree	Slightly disagree	Slightly agree	Partially agree	Completely agree	No Response
14. A consultation recording enhances the understanding of information.	☐	☐	☐	☐	☐	☐	☐
15. A consultation recording allows patients to retrospectively verify correct understanding of the information.	☐	☐	☐	☐	☐	☐	☐
16. A consultation recording allows patients to ensure that the physician has understood them correctly.	☐	☐	☐	☐	☐	☐	☐
17. A consultation recording allows patients to have a better recall of the information discussed.	☐	☐	☐	☐	☐	☐	☐
18. A consultation recording allows patients to prepare for follow-up appointments (.e.g. note down questions)	☐	☐	☐	☐	☐	☐	☐
19. A consultation recording improves the quality of communication.	☐	☐	☐	☐	☐	☐	☐

20. I am concerned that the quality of the communication decreases through a consultation recording.	☐	☐	☐	☐	☐	☐	☐
21. A consultation recording allows physicians to be more responsive of concerns and needs of patients.	☐	☐	☐	☐	☐	☐	☐
22. I am concerned that patients would be reserved and less open if consultations were recorded.	☐	☐	☐	☐	☐	☐	☐
23. I am concerned that physicians would be reserved and less open if consultations were recorded.	☐	☐	☐	☐	☐	☐	☐
24. I am concerned that patients over-interpret statements made by the physician on the recording.	☐	☐	☐	☐	☐	☐	☐
25. A consultation recording improves the trust between patients and physicians.	☐	☐	☐	☐	☐	☐	☐
26. I am concerned that the trust between patients and physicians would decrease if consultations were recorded.	☐	☐	☐	☐	☐	☐	☐
27. I am concerned that the physician-patient-relationship would be more formal if consultations were recorded.	☐	☐	☐	☐	☐	☐	☐
28. I am concerned that a consultation recording would put pressure on physicians.	☐	☐	☐	☐	☐	☐	☐
29. A consultation recording facilitates an equal collaboration between patient and physician.	☐	☐	☐	☐	☐	☐	☐
30. A consultation recording facilitates patients' active and self-responsible managing of their disease.	☐	☐	☐	☐	☐	☐	☐
31. A consultation recording allows patients to compare their treatment options and make the best decision.	☐	☐	☐	☐	☐	☐	☐
32. A consultation recording encourages patients to engage with their diagnosis.	☐	☐	☐	☐	☐	☐	☐
33. I am concerned that a consultation recording puts too much responsibility on patients.	☐	☐	☐	☐	☐	☐	☐
34. I am concerned that listening to the consultation recording would be a psychological burden for patients.	☐	☐	☐	☐	☐	☐	☐
35. A consultation recording allows patients to share information with other health care professionals.	☐	☐	☐	☐	☐	☐	☐
36. A consultation recording allows patients to share information with their relatives.	☐	☐	☐	☐	☐	☐	☐
37. A consultation recording allows relatives to provide better support to the patient.	☐	☐	☐	☐	☐	☐	☐
38. I am concerned that relatives could pressure patients into allowing them to listen to their consultation recording.	☐	☐	☐	☐	☐	☐	☐
39. A consultation recording provides evidence of what was said and done.	☐	☐	☐	☐	☐	☐	☐
40. A consultation recording provides a protection for patients and physicians.	☐	☐	☐	☐	☐	☐	☐
41. A consultation recording provides evidence in case of malpractice.	☐	☐	☐	☐	☐	☐	☐
42. I am concerned that a consultation recording would be used as evidence against physicians.	☐	☐	☐	☐	☐	☐	☐
43. I am concerned what happens with the consultation recording.	☐	☐	☐	☐	☐	☐	☐
44. I am concerned about confidentiality and data protection if consultations were recorded.	☐	☐	☐	☐	☐	☐	☐

45.	I am concerned that consultation recordings could be passed on undesirably.	☐	☐	☐	☐	☐	☐	☐
46.	I am concerned that the recording of the consultation could be distorted.	☐	☐	☐	☐	☐	☐	☐
47.	A consultation recording is helpful for treatment planning.	☐	☐	☐	☐	☐	☐	☐
48.	A consultation recording allows a better adherence to medical instructions.	☐	☐	☐	☐	☐	☐	☐
49.	I am concerned that a consultation recording disrupts clinical routines.	☐	☐	☐	☐	☐	☐	☐
50.	I am concerned that the technical requirements for making consultation recordings don't exist.	☐	☐	☐	☐	☐	☐	☐
51.	I am concerned that recording consultations is too complicated for physicians.	☐	☐	☐	☐	☐	☐	☐
52.	I am concerned that recording consultations is too complicated for patients.	☐	☐	☐	☐	☐	☐	☐
53.	I am concerned about patients perceiving a recording device as stressful during consultations.	☐	☐	☐	☐	☐	☐	☐
54.	I am concerned that consultation recordings prolong consultations.	☐	☐	☐	☐	☐	☐	☐
55.	A consultation recording reduces consultation length.	☐	☐	☐	☐	☐	☐	☐
56.	A consultation recording is especially helpful for older people.	☐	☐	☐	☐	☐	☐	☐
57.	A consultation recording is especially helpful for people with language barriers.	☐	☐	☐	☐	☐	☐	☐
58.	A consultation recording is especially helpful for people with cognitive deficits.	☐	☐	☐	☐	☐	☐	☐
59.	A consultation recording is especially helpful when starting or changing a treatment.	☐	☐	☐	☐	☐	☐	☐
60.	A consultation recording is especially helpful when treatments are complex and extensive.	☐	☐	☐	☐	☐	☐	☐
61.	A consultation recording is especially helpful in consultations in which treatment decisions are made.	☐	☐	☐	☐	☐	☐	☐
62.	A consultation recording should also be conducted when the diagnosis is communicated during the consultation.	☐	☐	☐	☐	☐	☐	☐
63.	A consultation recording should be made even in brief consultations with little amount of new information.	☐	☐	☐	☐	☐	☐	☐

Desire for the provision of audio recordings of medical encounters

The following questions pertain to your potential preferences regarding the provision of audio recordings of your own medical encounters.

64. In the future, would you like to provide audio recordings of your medical consultations to your patients?

☐ Yes ☐ No ☐ Maybe

65. *[If future desire „yes" or „maybe":]* Should the audio recording also be available to you?

☐ Yes ☐ No

66. *[If future desire „yes" or „maybe":]* Would you be willing to listen to such a recording after the consultation?

☐ Yes ☐ No ☐ Yes, if…*(please specify):* ________________________

67. *[If future desire „yes" or „maybe":]* Would you be open to patients making these consultation recordings on their cell phone?

☐ Yes ☐ No

68. **Would you be interested in recording the consultation if you or a family member were the patient?**

☐ Yes ☐ No ☐ Maybe

Your desire for participation

69. **Who, in your opinion, should make treatment decisions?**

Please select the statement that most closely aligns with your attitude.

Patients should make the decision what medical treatment they receive	Patients should ultimately make the decision about their medical treatment, after having seriously considered my medical opinion.	I prefer my patient and I to share the responsibility for making the decision which medical treatment is best for them.	I prefer to make the final decision about the patient's medical treatment, considering their opinion.	As a doctor, I prefer to make all decisions concerning the patient's medical treatment.
☐	☐	☐	☐	☐

Affinity for Technology Interaction

In the following questionnaire, we will ask you about your interaction with technical systems. The term "technical systems" refers to apps and other software applications, as well as entire digital devices (e.g., mobile phone, computer, TV, car navigation).

Please indicate the degree to which you agree/disagree with the following statements.

	Completely disagree	Largely disagree	Slightly disagree	Slightly agree	Completely agree	Completely agree
70. I like to occupy myself in greater detail with technical systems.	☐	☐	☐	☐	☐	☐
71. I like testing the functions of new technical systems	☐	☐	☐	☐	☐	☐
72. It is enough for me that a technical system works; I don't care how or why.	☐	☐	☐	☐	☐	☐
73. It is enough for me to know the basic functions of a technical system.	☐	☐	☐	☐	☐	☐

Knowledge about law

74. **Do you know the laws that regulate audio recordings of conversations in Germany?** ☐ Yes ☐ No ☐ No response

Questions about your person

Please provide some information about yourself below.

75. **Age in years** ☐ Younger than 18 years ☐ 18-29 years ☐ 30-39 years ☐ 40-49 years ☐ 50-59 years ☐ 60-69 years ☐ 70-79 years ☐ 80 years and older

76. **Gender** ☐ Female ☐ Male ☐ Non binary/diverse

77. **Education level**

☐ In residency training ☐ Completed residency

78. **I work as…**

☐ Junior physician at a hospital ☐ Senior or head physician at a hospital ☐ Self-employed in own outpatient practice

☐ Employee in an outpatient practice ☐ Other

79. **Work experience in oncology**

☐ Less than 5 years ☐ 5-10 years ☐ 11-20 years ☐ More than 20 years

80. **In which federal state do you work?**

- ☐ Schleswig-Holstein
- ☐ Hamburg
- ☐ Lower Saxony
- ☐ Bremen
- ☐ North Rhine-Westphalia
- ☐ Hesse
- ☐ Rhineland-Palatinate
- ☐ Baden-Württemberg
- ☐ Bavaria
- ☐ Saarland
- ☐ Berlin
- ☐ Brandenburg
- ☐ Mecklenburg-Western Pomerania
- ☐ Saxony
- ☐ Saxony-Anhalt
- ☐ Thuringia

SUPPLEMENTARY FILE 4

Table

Distribution of invitation for online survey

Way of distribution	N
**Contacted by email:** Number of clinics/groups contacted…	
Oncological outpatient clinics including all cancer centers and their collaborating clinics (nationwide)	559
Gynecological outpatient clinics nationwide	67
Working groups of the German Society for Haematology and Oncology (DGHO)	57
Inpatient and outpatient clinics at the UKE[a]	10
**Distribution of invitation by email:** Number of invitations sent to…	
Press release of UKE[a]	1
Newsletter of the University Cancer Center of UKE[a]	1
Newsletter of the Psycho-Oncology Working Group of the German Cancer Society	1
**Posts on social media / websites:** Number of (re-)posts on…	
Social media (Twitter, LinkedIn)	37
Social media accounts of UKE[a] (Instagram, Twitter)	2
**Distribution of leaflets through mail:** Number of leaflets sent to…	
Oncological outpatient clinics nationwide	320
Oncological rehabilitation clinics nationwide	320
Gynecological outpatient clinics nationwide	40
**Distribution of leaflets at conferences:** Number of leaflets distributed at…	
German Cancer Congress 2022 (DKK)	70
Oncological Congress in North Germany (“Norddeutsches Onkologieforum”) 2023	50
20th Annual Congress of the Psycho-Oncology Working Group of the German Cancer Society 2022	40
Congress of the German Society for Haematology and Oncology 2023 (DGHO)	30

Notes: [a] UKE = University Medical Center Hamburg-Eppendorf;
